# 620. Development and Validation of a Novel qPCR Assay for Differentiation of Common *Candida* Pathogens including *C. auris*

**DOI:** 10.1093/ofid/ofad500.686

**Published:** 2023-11-27

**Authors:** Clayton Thomas, James Grantham, Emily Lute, Jerod Davidson, Scott Cowden, Jamie Nutt

**Affiliations:** Eurofins Viracor, Lenexa, Kansas; Eurofins Viracor, Lenexa, Kansas; Eurofins Viracor, Lenexa, Kansas; Eurofins Viracor, Lenexa, Kansas; Eurofins Viracor Clinical Diagnostics, Lenexa, Kansas; Eurofins Viracor, Lenexa, Kansas

## Abstract

**Background:**

*Candida* is a common asymptomatic fungal colonizer of humans. Of the 200+ total species in the genus *Candida*, only a few are known to cause infection. The most common are *C. albicans*,* C. glabrata*, *C. parapsilosis*,* C. tropicalis*, *C. krusei*, and *C. auris*. In the USA, candidemia is the fourth most common cause of bloodstream infections in hospitalized patients. Although antifungal medication can treat candidemia, mortality rates remain between 30 and 60%. Eurofins Viracor has developed a qPCR assay that will qualitatively detect and differentiate any of the 6 previously listed *Candida* species in whole blood. Speciation is critical for treatment due to variable drug susceptibility among different species, particularly *C. auris* due to its multi-drug resistance.

**Methods:**

Multiple primer/probe mixes were designed around and within the ITS1, 5.8s, ITS2, and 28s ribosomal subunit regions in each of the six *Candida* target species. The 5.8s and 28s regions are heavily conserved, while the ITS regions are highly divergent and used to differentiate species. After optimization, the six primer/probe mixes were partially multiplexed resulting in four mixes covering all species. DNA was extracted from human blood spiked with viable *Candida* using the MagMAX™ DNA Multi-Sample Ultra 2.0 Kit, and eluates were amplified using the Applied Biosystems™ 7500 Fast platform.

**Results:**

Each assay met all validation acceptance criteria and demonstrated excellent accuracy, precision, linearity and dynamic range, and sensitivity. 32 non-*Candida* fungal and bacterial pathogens that commonly cause bloodstream infections were used to assess specificity. All specificity samples were negative for all *Candida* targets. To assess accuracy, 75 of 75 *Candida* samples representing all six species across a range of concentrations were tested and correctly identified. 24 of 25 known-negative samples were negative, with one positive *C. glabrata* C_T_ result observed just below the cutoff.

**Conclusion:**

Traditional blood culture methods for *Candida* species identification can be too time consuming for a quickly progressing infection. The *Candida* qPCR assay described here not only detects an infection faster but provides accurate speciation allowing for rapid and targeted antifungal treatment options for the patient.

**Disclosures:**

**Clayton Thomas, BS in Biology**, Eurofins Viracor: Employee **James Grantham, n/a**, Eurofins Viracor: Employee **Emily Lute, n/a**, Eurofins Viracor: Employee **Jerod Davidson, n/a**, Eurofins Viracor: Employee **Scott Cowden, MS, MB ASCP(CM)**, Eurofins Viracor: Employee **Jamie Nutt, n/a**, Eurofins Viracor: Employee

